# ALMS1 Regulates TGF-β Signaling and Morphology of Primary Cilia

**DOI:** 10.3389/fcell.2021.623829

**Published:** 2021-02-01

**Authors:** María Álvarez-Satta, Mauro Lago-Docampo, Brais Bea-Mascato, Carlos Solarat, Sheila Castro-Sánchez, Søren T. Christensen, Diana Valverde

**Affiliations:** ^1^CINBIO, Universidade de Vigo, Vigo, Spain; ^2^Instituto de Investigación Sanitaria Galicia Sur (IIS Galicia Sur), Hospital Álvaro Cunqueiro, Vigo, Spain; ^3^Department of Biology, Section of Cell Biology and Physiology, The August Krogh Building, University of Copenhagen, Copenhagen, Denmark

**Keywords:** ciliopathies, Alström syndrome (AS), ALMS1, primary cilium, TGF-β/BMP signaling, ciliary length, ciliary morphology, hTERT RPE-1 cells

## Abstract

In this study, we aimed to evaluate the role of ALMS1 in the morphology of primary cilia and regulation of cellular signaling using a knockdown model of the hTERT-RPE1 cell line. ALMS1 depletion resulted in the formation of longer cilia, which often displayed altered morphology as evidenced by extensive twisting and bending of the axoneme. Transforming growth factor beta/bone morphogenetic protein (TGF-β/BMP) signaling, which is regulated by primary cilia, was similarly affected by ALMS1 depletion as judged by reduced levels of TGFβ-1-mediated activation of SMAD2/3. These results provide novel information on the role of ALMS1 in the function of primary cilia and processing of cellular signaling, which when aberrantly regulated may underlie Alström syndrome.

## Introduction

Alström syndrome (ALMS; MIM#203800) is a rare, autosomal recessive disorder included in the group of ciliopathies, which share a common etiology: defects in the structure and function of cilia (widely reviewed in Mitchison and Valente, 2017; Reiter and Leroux, 2017). The clinical spectrum displayed by ALMS patients includes: early-onset retinal dystrophy, obesity with type 2 diabetes mellitus (T2DM), hyperinsulinemia and insulin resistance, sensorineural hearing loss, dilated cardiomyopathy, renal, hepatic and pulmonary injury, and widespread fibrosis (reviewed in Marshall et al., 2011). As far as we know, ALMS represents a particular entity within ciliopathies since, it is a monogenic syndrome caused by mutations in the *ALMS1* gene (HGNC:428). This gene encodes a protein of 4,169 amino acids localized to centrosomes and basal bodies of primary cilia (Collin et al., 2002; Hearn et al., 2002, 2005).

The ALMS1 protein plays a broad range of biological roles, including cilia maintenance and function, endosomal trafficking, control of cell cycle, cellular differentiation, and energy balance homeostasis (reviewed in Girard and Petrovsky, 2011; Álvarez-Satta et al., 2015; Hearn, 2019). While some studies showed that ALMS1 is required for primary cilium formation and centrosomal cohesion (Graser et al., 2007; Li et al., 2007; Knorz et al., 2010), others report no essential role in ciliogenesis (Collin et al., 2005, 2012; Hearn et al., 2005; Chen et al., 2017).

Despite great progress has been made to determine the ciliary and extraciliary roles from ALMS1, the molecular basis underlying ALMS is still largely unknown. Therefore, it seems possible that this protein is involved in other cellular pathways still unexplored. Interestingly, cardiac anomalies and multiorgan fibrosis, common phenotypes related to ALMS, are well-known to be associated with aberrant transforming growth factor beta/bone morphogenetic protein (TGF-β/BMP) signaling (reviewed in Heger et al., 2016; Walton et al., 2017). The TGF-β/BMP pathway acts as a central regulator of cell proliferation, differentiation, and survival programs, usually triggering anti-inflammatory and cytostatic responses (reviewed in Massagué, 2012; Zhang et al., 2017). This pathway operates in tightly spatiotemporal coordination with many other signaling cascades such as Wnt, Hippo, Notch, Hedgehog (Hh), mitogen-activated protein kinase (MAPK), or phosphoinositide 3-kinase (PI3K)-Akt, representing a central partner of extensive crosstalk that will eventually determine the specific effect in different cell types ans conditions (Luo, 2017).

The TGF-β/BMP pathway can activate two types of cascades when stimulated: canonical and non-canonical (Massagué, 2012). The canonical signaling is mediated by specific transcription factors called R-SMAD proteins (SMAD2/3 in TGF-β/activin/nodal signaling and SMAD1/5/8 in BMP signaling), whereas the non-canonical response comprises a plethora of non-R-SMAD pathways that complement SMAD action. They include cascades mediated by Rho GTPases or MAPKs such as the extracellular signal-regulated protein kinases 1 and 2 (ERK1/2), and also the PI3K-Akt-mTOR pathway, among others.

Remarkably, the primary cilium is known to be a key regulator of TGF-β/BMP signaling by clathrin-dependent endocytosis (CDE) at ciliary pocket, an invagination of the plasma membrane at the base of the cilium that represents a major site for exo- and endocytosis (reviewed in Pedersen et al., 2016; Christensen et al., 2017). Thus, TGF-β receptors and other components of the signaling machinery localize to the ciliary pocket of primary cilia (Clement et al., 2013; Vestergaard et al., 2016; Gencer et al., 2017). Besides, SMAD2/3 and ERK1/2 phosphorylation and subsequent activation occur at this ciliary domain (Clement et al., 2013), supporting the idea that primary cilia are important for balancing the output of TGF-β/BMP signaling.

Therefore, it is necessary to further explore the molecular basis of ALMS to decipher the pathophysiological mechanisms underlying ALMS phenotypes and also to better define the involvement of ALMS1 in the ciliary activity. In this study, we aimed to establish an *ALMS1* knockdown model from cultured mammalian cells to analyze the effect of ALMS1 depletion on: (i) primary cilia, assessing ciliary length, and morphology, and (ii) TGF-β/BMP signaling, performing stimulation assays to find a possible molecular link between ALMS and the TGF-β/BMP pathway.

## Materials and Methods

### Cell Culture and Transfection

hTERT RPE-1 (RPE-1) and HeLa cells [laboratory stock, derived from American Type Culture Collection (ATCC) clone CRL-4000] were cultured in Dulbecco's modified Eagle's medium (DMEM, Sigma-Aldrich) supplemented with 10% fetal bovine serum (FBS, PAA laboratories), 1% penicillin-streptomycin (Gibco), and 2 mM L-Glutamine (Lonza) at 37°C and 5% CO_2_ atmosphere in a humidified incubator. Cultures were passaged once or twice a week. For *ALMS1* silencing, RPE-1 cells were transiently transfected using a commercial pool of four small interfering RNAs (siRNAs) [ON-TARGET plus Human ALMS1 (7840) SMARTpool (5 nmol) from Dharmacon]. A negative control siRNA (5′-UAAUGUAUUGGAACGCAUA-3′, Eurofins Genomics) was assayed in parallel for each experiment. We followed a reverse transfection protocol using Lipofectamine®RNAiMAX (Invitrogen) according to the manufacturer's instructions and maintaining a final concentration of 30 nM siRNA. For that, cells were plated on coverslips or cultured dishes from a 90 to 95% confluent flask to reach 30–50% confluency at 24 h after plating. Then, the culture medium was changed to a serum-free medium, and cells were incubated for additional 48 h before harvesting to promote primary cilia formation.

### Quantitative RT–PCR

Total RNA was extracted from cells using Nucleospin®RNA (Macherey-Nagel) according to the manufacturer's protocol. A total of 790–850 ng of RNA was reverse transcribed into cDNA using SuperScript® IV Reverse Transcriptase (Invitrogen) with an incubation step at 50°C during 50 min as recommended for long transcripts such as *ALMS1*. Relative quantification was carried out in 20 μL reactions using 2 μL of diluted cDNA [1:50 for the reference gene beta-actin (*ACTB*) and 1:10 for *ALMS1* gene] and 1 μL of commercial TaqMan® Gene Expression Assays (Hs01060665_g1 and Hs00367316_m1, respectively). All samples were run in triplicate on the StepOne™ Real-Time PCR System (Applied Biosystems) for 10 min at 95°C, 40 cycles of 15 s at 95°C and 1 min at 60°C. The relative amount of *ALMS1* mRNA normalized to *ACTB* was calculated in each sample using the Pfaffl model (Pfaffl, 2001). Biological replicates were repeated three times per condition (control and siRNA-treated cells).

### Immunofluorescence Microscopy Analysis

Cells grown on glass coverslips were washed twice in cold PBS and fixed in a cold 4% (w/v) paraformaldehyde (PFA) solution for 15 min, followed by two washes with PBS and a permeabilization step using PBS/0.2% (v/v) Triton-X100/1% (w/v) bovine serum albumin (BSA) buffer for 12 min. After one PBS wash, cells were subjected to double immunostaining starting with a block step in a PBS/2% (w/v) BSA solution for 30 min at room temperature (RT), then incubation with primary antibodies in blocking solution for 90 min at RT, three washes with PBS for 5 min each, incubation with secondary antibodies and DAPI (1 μg/mL) in blocking solution for 45 min at RT in the dark, and finally, three washes with PBS for 5 min each. Once finished, coverslips were mounted in Prolong® Diamond Antifade Mountant (Molecular Probes) according to manufacturer's instructions and imaged at 100× magnification using an Olympus BX-83 upright microscope with a DP71 color camera with DP controller software. Images were processed for publication using Adobe Photoshop (v. CS6) or Fiji/ImageJ software (v.1.51d). Measurement of cilia length was carried out on imaged cells in at least 50 cells per condition across three-independent experiments. The following primary antibodies and dilutions were used: rabbit anti-ALMS1 (Abcam, ab84892, 1:1,000) and mouse anti-acetylated tubulin (Sigma-Aldrich, T6793 clone 6-11b-1, 1:2,000) as a ciliary marker. Secondary antibodies were used at 1:600: Alexa Fluor® 488-conjugated goat anti-rabbit (Thermo Scientific, A-11008) and Alexa Fluor® 594-conjugated goat anti-mouse (Thermo Scientific, A-11005). Nuclei were stained with DAPI.

### Ligand Stimulation Assays

For TGF-β/BMP stimulation assays, control, and siRNA-treated cells were washed once in commercial DPBS (Sigma-Aldrich) and incubated in fresh DMEM with 2 mM L-Glutamine for 30 min before stimulation. Then, TGFβ and BMP signaling were independently stimulated by adding specific ligands for 0, 10, 30, and 90 min: recombinant human TGFβ1 (rhTGF-β1; R&D Systems, 240-B) at 2 ng/mL (final concentration) and recombinant human/mouse/rat BMP-2 (rh/m/rBMP2; R&D Systems, 355-BM) at 50 ng/mL. After stimulation, cells were washed twice in ice-cold PBS, harvested using a scraper, and centrifuged in a Sigma® 1–14 K at 4°C, 7,400×*g* for 10 min. Then, pellets were incubated on ice for 10 min in 40–50 μL of ice-cold RIPA lysis buffer with 0.1% (v/v) protease inhibitor cocktail (Calbiochem) and 1 mM sodium orthovanadate as phosphatase inhibitor (Sigma-Aldrich). Lysates were centrifuged at 4°C, 14,500×*g* for 25 min, and the supernatant was collected and conserved at −20°C until used. Protein concentration was measured by Bradford assay (Bradford, 1976) with the Bio-Rad protein assay.

For the *ALMS1* TGF-β/BMP stimulation assay, we starved HeLa cells for 24 h, and then added 2/4 ng/mL TGF-β or 50/100 ng/mL BMP. We extracted mRNA before stimulation and 24 h after stimulation.

### SDS-PAGE, Western Blotting, and Quantification

Samples for SDS-PAGE were prepared by mixing 20 μg of each lysate with Laemmli sample buffer (Bio-Rad) and 5% (v/v) β-mercaptoethanol, then boiled at 95°C for 5 min and run on a 12% Mini-PROTEAN® TGX™ precast gel (Bio-Rad) for 40–50 min. Proteins were transferred onto a polyvinylidene fluoride (PVDF) membrane using the Trans-Blot® Turbo™ Transfer System (Bio-Rad) at 25 V for 30 min. Membranes were blocked in 5% (w/v) non-fat dry milk in TBST [TBS/0.1% (v/v) Tween-20] for 1.5 h at RT, then incubated overnight at 4°C with primary antibodies diluted in blocking solution, washed three times for 5 min each with blocking solution, incubated with horseradish peroxidase-conjugated secondary antibodies diluted in blocking solution for 45 min at RT and finally washed three times for 5 min each with TBS.

Blots were revealed using Clarity™ Western ECL Substrate (Bio-Rad) in a ChemiDoc™ XRS+ System and Image Lab™ software (v.3.0, Bio-Rad). Blots were probed for detection of phosphorylated SMAD2 (pSMAD2; approximately 60 kDa), phosphorylated SMAD1/5 (pSMAD1/5; 60 kDa),and phosphorylated ERK1/2 (pERK1/2; 42, 44 kDa). Immunoblots signals were quantified by densitometry analysis using Image Lab™: each intensity value was normalized to total protein staining as a loading control (following a standard protocol for Coomassie Brilliant Blue R-250 staining) and then fold-change ratios were calculated using the corresponding normalized intensity value of the control sample stimulated for 30 min as a reference in each blot. At least three biological replicates from separate experiments were used for protein level calculations. The following primary antibodies and dilutions were used: rabbit anti-phospho-SMAD2 (3101, 1:500), rabbit anti-phospho-Smad1/5 (9516, 1:500), and rabbit anti-phospho-p44/42 MAPK (ERK1/2) (9101, 1:500) from Cell Signaling Technology. Anti-rabbit IgG HRP-linked (Sigma-Aldrich, A0545) was used as secondary antibody with 1:15,000 dilution,).

### Statistical Analysis

All data are presented as mean ± standard deviation (SD). Initially, a Shapiro test was carried out to confirm the normality of the data. To compare the calculated means for ciliary length between control and siRNA-treated cells, we used the Student's *t*-test for two independent samples, while for protein levels we used a two-way ANOVA with Sidak *post-hoc* correction. All tests were considered statistically significant if *p* ≤ 0.05. Statistical analyses were accomplished with the SPSS software (v.15.0, SPSS Inc.) or GraphPad Prism (version 7.00 for Windows, GraphPad Software).

## Results

### ALMS1 Knockdown Affects Ciliary Length and Morphology in hTERT RPE-1 Cells

**Figure 1:**
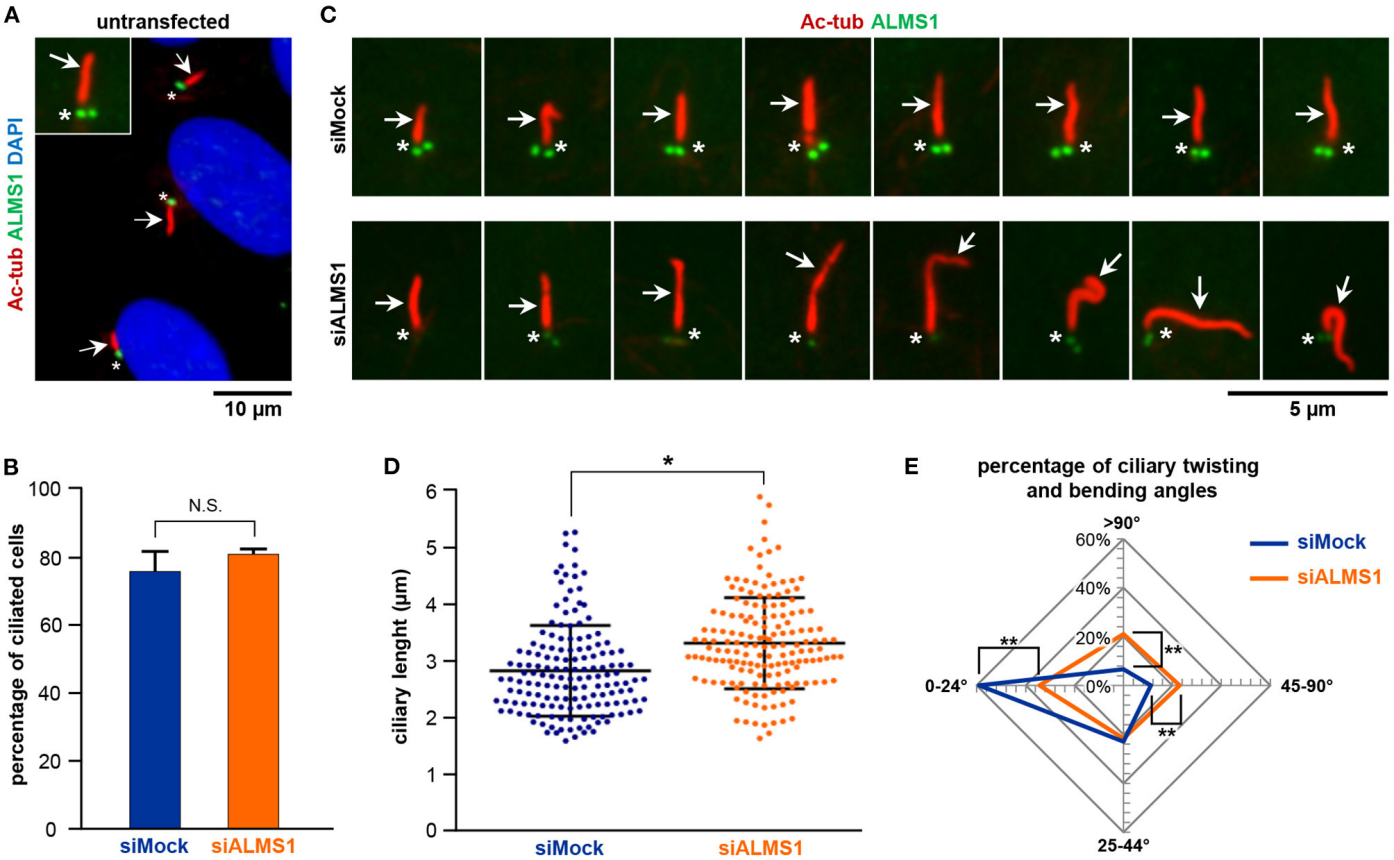
ALMS1 knockdown increases the number of aberrant cilia and ciliary length. **(A)** Representative immunofluorescence image of primary cilia in untransfected cells. Primary cilia (arrows) are stained in red, ALMS1 protein (asterisks) in green and cell nuclei in blue (DAPI). A zoomed cilium is also shown. **(B)** Percentage of ciliated cells in control (siMock) and silenced (siALMS1) cells (*n* = 3). **(C)** Representative immunofluorescence images of primary cilia in control and silenced cells showing different aberrant morphologies (*n* = 3). **(D)** Dot plot representing each of the measurements of ciliary length for control and silenced cells; *n* = 3 with at least 50 cilia measured per biological replicate. **(E)** Radar plot of the percentage of ciliary twisting and its bending angles between control and silenced cells (*n* = 3). Error bars represent mean ± SD (*p < 0.05, **p < 0.01).

To initially verify ALMS1 localization to the base of primary cilia, RPE1 cells were depleted for a serum for 24 h to induce growth arrest followed by immunofluorescence microscopy analysis (IFM). As shown in Figure 1A, ALMS1 predominantly localizes to the ciliary base and often as two puncta, which correspond to the centrioles of the centrosome (Hearn et al., 2005). Our analysis indicated no localization of the protein to extra-ciliary sites, suggesting that ALMS1 is mostly confined to the ciliary base in growth-arrested RPE1 cells as previously reported (Hearn et al., 2005). Next, to investigate if ALMS1 plays a role in cilia formation, RPE-1 cells were transfected with a pool of commercially available siRNAs against *ALMS1* gene and 72 h after transfection, which included 24 h of serum starvation, depletion efficiency was assessed by RT-qPCR and IFM analyses. A mean reduction of 67% (mean ± SD: 66.83 ± 3.72%; *n* = 3) in *ALMS1* expression was achieved (Supplementary Figure 1A), and as judged by IFM the level of ALMS1 was greatly reduced at the cilia base, confirming knockdown at the protein level (Supplementary Figure 1B).

While depletion of ALMS1 did not affect the ability of cells to form primary cilia (Figure 1B), we observed that siRNA-treated cells displayed significantly longer cilia compared to mock-treated cells (mean ± SD: 3.28 ± 0.18 μm in siRNA-treated cells vs. 2.83 ± 0.06 μm in controls; *p* = 0.041) (Figures 1C,D). Further, we noticed that ALMS1-depleted cells in many cases presented cilia with abnormal morphologies, including axonemal segmentation, ciliary bulging, and most prominently extensive ciliary bending and/or twisting, which occasionally presented as zigzag folding along the ciliary axis (Figure 1C). The number of cilia with twisted configuration was evaluated by measuring the angle of ciliary bending along the axoneme: 0–24°, 25–44°, 45–90°, and >90°. As indicated in the radar plot (Figure 1E), ALMS1-depleted cells presented significantly more cells with ciliary bending 45–90° (mean ± SD: 23.6 ± 1.2% in siRNA-treated cells vs. 11.2 ± 1.7% in controls; *p* = 0.002) and >90° (mean ± SD: 21.0 ± 3.1% in siRNA-treated cells vs. 6.7 ± 2.7% in controls; *p* = 0.003). A significantly lower number of cells subjected to ALSM1 knockdown displayed cilia with no or little bending (0–24°) (mean ± SD: 33.7 ± 1.3% in siRNA-treated cells vs. 59.1 ± 6.6% in controls; *p* = 0.008).

### ALMS1 Depletion Leads to Downregulation of TGF-β Signaling

**Figure 2:**
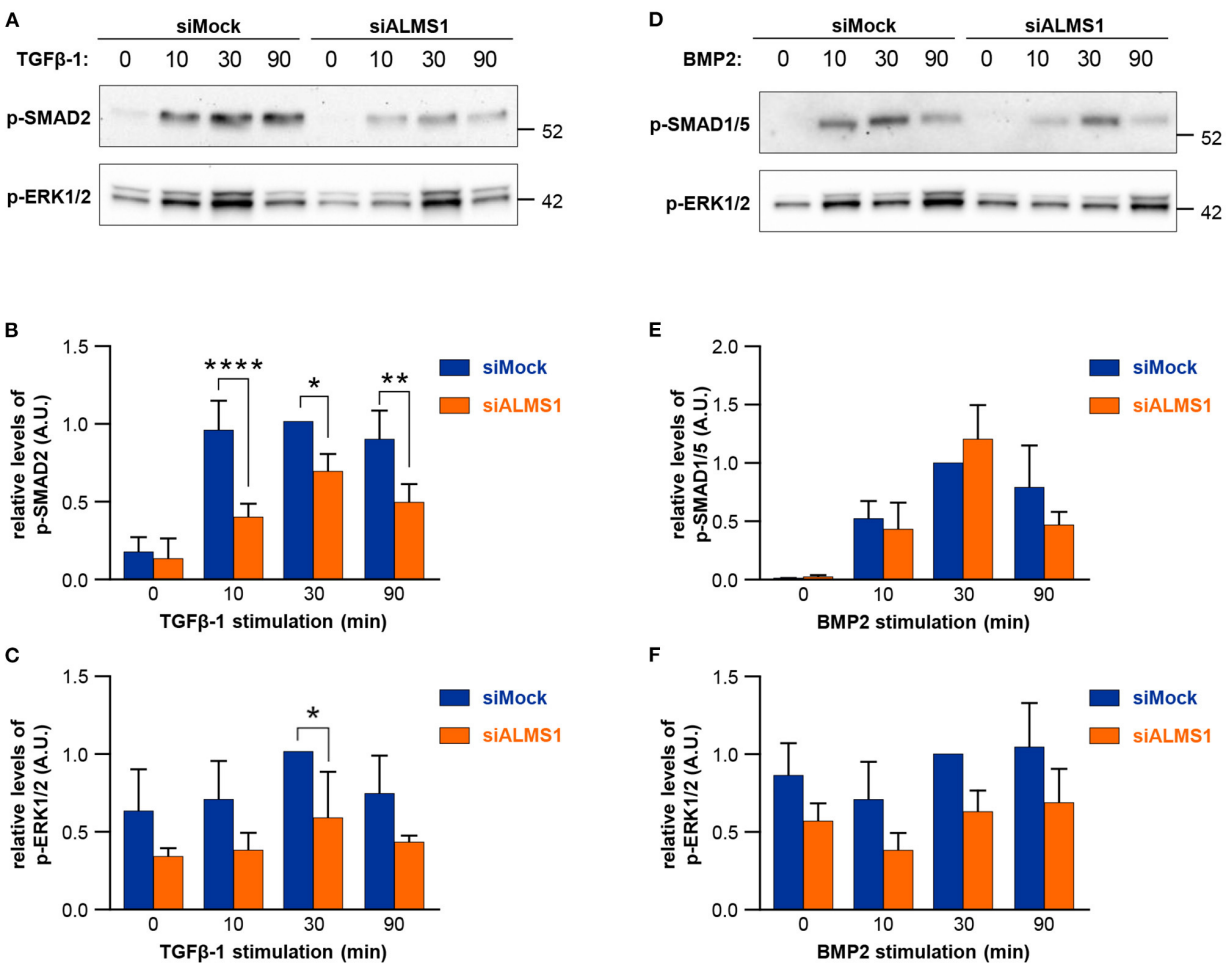
Transient ALMS1 knockdown is associated with a decrease in SMAD2 phosphorylation following TGF-β1 stimulation and a decrease in ERK1/2 phosphorylation after BMP-2 treatment. **(A)** Representative immunoblot for p-SMAD2 and p-ERK1/2 proteins in control (siMock) and silenced (siALMS1) RPE-1 cells after TGF-β1 stimulation at 0, 10, 30, and 90 min (*n* = 4). **(B)** Bar plot of the pSMAD2 normalized protein levels after TGF-β1 stimulation (*n* = 4). **(C)** Bar plot of the normalized p-ERK1/2 protein levels after TGF-β1 stimulation (*n* = 4). **(D)** Representative immunoblot for p-SMAD1/5 and p-ERK1/2 proteins in control (siMock) and silenced (siALMS1) RPE-1 cells after BMP-2 stimulation at 0, 10, 30, and 90 min (*n* = 3). **(E)**. Bar plot of the pSMAD1/5 normalized protein levels after BMP-2 stimulation (*n* = 3). **(F)** Bar plot of the normalized pERK1/2 protein levels after BMP-2 stimulation (*n* = 3). All protein quantification data are presented as Arbitrary Units (AU) (*p < 0.05, **p < 0.01, ****p < 0.0001).

In TGFβ-1 stimulated cells, we observed a significant reduction in activation of SMAD2/3 at all time-points of stimulation in ALMS1-depleted cells (10 min: *p* < 0.0001, 30 min: *p* = 0.0149 and 90 min: *p* = 0.0028) and ERK1/2 at 30 min (*p* = 0.027) (Figures 2A–C). In contrast, BMP-2-mediated activation of SMAD1/5 and ERK1/2 was largely unaffected in ALMS1-depleted cells (Figures 2D–F).

### ALMS1 mRNA Levels Increase After TGFβ-1 and BMP2 Stimulation

**Figure 3:**
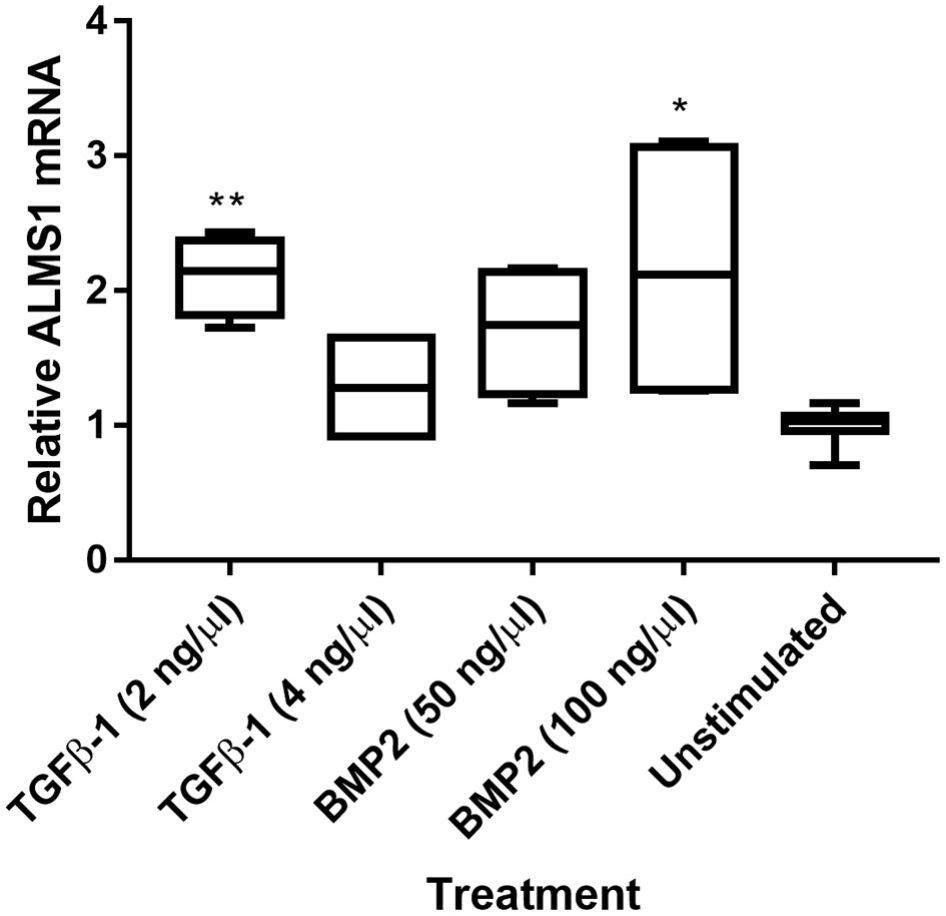
*ALMS1* expression is enhanced upon stimulation with TGF-B/BMP in HeLa cells. Treatments with TGFβ-1 (2 ng/Ml, *p* = 0.0073, *n* = 2) and BMP2 (100 ng/mL, *p* = 0.02, *n* = 2) showed statistical significance after a Kruskal-Wallis test with Dunn's correction in comparison with the unstimulated cells (*n* = 4). *p < 0.05, **p < 0.01.

We extracted mRNA after ligand stimulation to asses the effect of the predicted SMAD-binding sites within *ALMS1* promoter (Supplementary Table 1). Our qPCR results showed that *ALMS1* levels increase after the stimulation of both TGFβ-1 (2 ng/Ml, *p* = 0.0073, *n* = 2) and BMP2 (100 ng/mL, *p* = 0.02, *n* = 2) (Figure 3).

## Discussion

The study of ALMS has generated much interest in the last years since it represents a monogenic model of obesity and metabolic syndrome. Nevertheless, the molecular basis of ALMS has not yet been elucidated. ALMS1 protein plays a wide range of biological roles, encompassing both ciliary and extraciliary functions, what has raised some controversy about ALMS should be considered a ciliopathy caused by cilia dysfunction or if, however, it is associated with a loss of ALMS1 functions unrelated to primary cilia (Chen et al., 2017; Hearn, 2019). Several hallmark features of ALMS such as cardiomyopathy, widespread fibrosis, and severe obesity with metabolic syndrome are known to be related to deregulation of TGF-β/BMP signaling (Zamani and Brown, 2011; Grgurevic et al., 2016; Heger et al., 2016; Walton et al., 2017); however, the possible connection between this pathway and the ALMS1 protein has not been extensively studied.

Here we report a role for *ALMS1* gene in primary cilia assembly, affecting both ciliary length and morphology, in an RPE-1 model. Upon loss of *ALMS1* expression, we observed that depleted cells assembled longer cilia with a higher proportion of aberrant morphologies. Interestingly, a similar trend can be inferred from measurements in fibroblasts from several ALMS patients, who assembled slightly longer cilia (Chen et al., 2017). The formation of longer cilia has also been associated with ciliary proteins such as BBS4 and MKS1-3 in cultured cells from *Bbs4*^−/−^ model mice (Mokrzan et al., 2007) and Meckel patients (Tammachote et al., 2009). Thus, an abnormal ciliary length may affect both cargo transport and position along the cilium, which could disturb the normal activity of signaling cascades regulated by primary cilia (Mokrzan et al., 2007; Tammachote et al., 2009). In addition, longer cilia would have a direct impact on cell-cycle progression, tightly related to ciliary assembly and disassembly (reviewed in Ishikawa and Marshall, 2011). This is consistent with longer cell cycles reported in fibroblasts from ALMS patients (Zulato et al., 2011), which suggests a role for ALMS1 in the regulation of cell-cycle duration.

The functional link between ciliogenesis, actin dynamics, and endocytic recycling has been well defined in previous works on BBS4, BBS6, and other cilia-related proteins (Kim et al., 2010; Hernandez-Hernandez et al., 2013). Since the interaction of ALMS1 with α-actinin, a cytoskeletal actin binding-protein, and other components of the endosome recycling pathway has already been reported (Collin et al., 2012; Favaretto et al., 2014), it seems reasonable that *ALMS1* might be a key regulator of the ciliary assembly *via* actin remodeling control. On the other hand, the aberrant morphologies reported here suggest that ALMS1 loss is accompanied by disruption of the normal axoneme architecture.

In addition to its role in ciliary assembly, our work provides the first evidence of the implication of ALMS1 in the TGF-β/BMP pathway. Our results suggest that ALMS1 depletion downregulates the TGF-β signaling, (probably) disturbing the signaling through canonical and non-canonical pathways. ALMS1 may therefore be a key regulator of TGF-β signaling, which could explain some of the most severe features developed by ALMS patients. Fibrosis, one of the major causes of morbidity and mortality in ALMS, is primarily driven by TGF-β ligands, especially activators of the SMAD2/3 axis, which is upregulated in many human fibrotic conditions (Walton et al., 2017). Our result is consistent with previous observations that SMAD2 depletion promotes fibrosis through the specific activation of SMAD3, well characterized as a key mediator of pro-fibrotic responses (Meng et al., 2010). In addition, some evidence from Zulato et al. (2011) support our results: (i) fibrosis in ALMS is a primarily defect due to *ALMS1* mutations and (ii) ALMS fibroblasts show greater responsiveness to TGF-β stimulation, which produces an excessive deposition of extracellular matrix components. Therefore, we propose that *ALMS1* silencing leads to the inhibition of SMAD2 phosphorilation, which may prevent the protector effect against fibrosis associated with this protein and also trigger the systemic fibrosis displayed by ALMS patients. On the other hand, downregulation of TGF-β signaling could also explain other hallmark features related to ALMS such as dilated cardiomyopathy. Thus, several patients with *ALMS1* mutations show mitogenic cardiomyopathy, a rare form of dilated cardiomyopathy defined by an uncontrolled proliferation of cardiomyocytes (Louw et al., 2014; Shenje et al., 2014). Considering that both TGF-β/BMP signalings usually keep a balance between anti-proliferative and proliferative responses, ALMS1 could be a key regulator of this pathway. A persistent mitotic activity could be triggered if this balance is disturbed as a consequence of ALMS1 depletion. The essential role of ALMS1 in terminal cardiomyocyte cell cycle arrest for the maturation of the mammalian heart supports this possibility (Shenje et al., 2014). We thereby propose that alteration of TGF-β/BMP balance would be a new potential pathophysiological mechanism underlying ALMS.

Although ALMS1 seems to regulate both canonical and non-canonical cascades in response to TGF-β stimulation, the presence of binding sites for SMAD3/4 in *ALMS1* promoter (Supplementary Table 1) may point out that ALMS1 specifically acts on R-SMADs. We confirmed that upon stimulation of TGF-B/BMP, *ALMS1* expression is enhanced (Figure 3). In addition, the *ALMS1* sequence harbors nuclear localization signals (Collin et al., 2002) that might anticipate a potential role in regulating the nuclear translocation of active SMAD complexes to activate TGF-β/BMP target genes. Another possibility is that ALMS1 could modulate the levels of available TGF-β/BMP receptors given its role in endocytosis and trafficking-related processes (Collin et al., 2012; Favaretto et al., 2014), which are essential mechanisms for modulating the activation of TGF-β/BMP signaling (reviewed in Balogh et al., 2013; Ehrlich, 2016; Pedersen et al., 2016).

In conclusion, we report a role for *ALMS1* in ciliary assembly and also in the inhibition of TGF-β signaling in cultured human cells, which may represent novel pathophysiological mechanisms underlying ALMS that could explain some of the most severe phenotypes related to this syndrome, such as fibrosis or cardiomyopathy. Despite our results are based on an *in vitro* system of cultured cells, the finding of TGF-β alterations related to ALMS1 might open new therapeutic options that should be explored for ALMS patients.

## Data Availability Statement

The raw data supporting the conclusions of this article will be made available by the authors, without undue reservation.

## Author Contributions

MÁ-S and DV designed the study. MÁ-S, ML-D, and BB-M performed the experiments. MÁ-S, ML-D, BB-M, CS, SC-S, and DV analyzed the data. MÁ-S, ML-D, BB-M, SC-S, and DV written and revised the manuscript. STC designed the study, analyzed the data and written, and revised the manuscript. All authors contributed to the article and approved the submitted version.

## Conflict of Interest

The authors declare that the research was conducted in the absence of any commercial or financial relationships that could be construed as a potential conflict of interest.

## References

[B1] Álvarez-Satta M. Castro-Sánchez S. Valverde D. (2015). Alström syndrome: current perspectives. App. Clin. Genet. 8, 171–179. 10.2147/TACG.S56612 26229500 PMC4516341

[B2] Balogh P. Katz S. Kiss A. L. (2013). The role of endocytic pathways in TGF-β signaling. Pathol. Oncol. Res. 19, 141–148. 10.1007/s12253-012-9595-8 23274761

[B3] Bradford M. M. (1976). A rapid and sensitive method for the quantitation of microgram quantities of protein utilizing the principle of protein-dye binding. Anal. Biochem. 72, 248–254. 10.1016/0003-2697(76)90527-3 942051

[B4] Chen J. H. Geberhiwot T. Barrett T. G. Paisey R. Semple R. K. (2017). Refining genotype-phenotype correlation in Alström syndrome through study of primary human fibroblasts. Mol. Genet. Genomic. Med. 5, 390–404. 10.1002/mgg3.296 28717663 PMC5511801

[B5] Christensen S. T. Morthorst S. K. Mogensen J. B. Pedersen L. B. (2017). Primary cilia and coordination of receptor tyrosine kinase (RTK) and transforming growth factor b (TGF-b) signaling. Cold Spring Harb. Perspect. Biol. 9:a028167. 10.1101/cshperspect.a028167 27638178 PMC5453389

[B6] Clement C. A. Ajbro K. D. Koefoed K. Vestergaard M. L. Veland I. R. Henriques de Jesus M. P.. (2013). TGF-β signaling is associated with endocytosis at the pocket region of the primary cilium. Cell Rep. 3, 1806–1814. 10.1016/j.celrep.2013.05.020 23746451

[B7] Collin G. B. Cyr E. Bronson R. Marshall J. D. Gifford E. J. Hicks W. (2005). Alms1-disrupted mice recapitulate human Alström syndrome. Hum. Mol. Genet. 14, 2323–2333. 10.1093/hmg/ddi235 16000322 PMC2862911

[B8] Collin G. B. Marshall J. D. Ikeda A. So W. V. Russell-Eggitt I. Maffei P.. (2002). Mutations in ALMS1 cause obesity, type 2 diabetes and neurosensory degeneration in Alström syndrome. Nat. Genet. 31, 74–78. 10.1038/ng867 11941369

[B9] Collin G. B. Marshall J. D. King B. L. Milan G. Maffei P. Jagger D. J.. (2012). The Alström syndrome protein, ALMS1, interacts with α-actinin and components of the endosome recycling pathway. PLoS ONE 7:e37925. 10.1371/journal.pone.0037925 22693585 PMC3365098

[B10] Ehrlich M. (2016). Endocytosis and trafficking of BMP receptors: regulatory mechanisms for fine-tuning the signaling response in different cellular contexts. Cytokine Growth Factor Rev. 27, 35–42. 10.1016/j.cytogfr.2015.12.008 26776724

[B11] Favaretto F. Milan G. Collin G. B. Marshall J. D. Stasi F. Maffei P.. (2014). GLUT4 defects in adipose tissue are early signs of metabolic alterations in Alms1GT/GT, a mouse model for obesity and insulin resistance. PLoS ONE 9:e109540. 10.1371/journal.pone.0109540 25299671 PMC4192353

[B12] Gencer S. Oleinik N. Kim J. Panneer Selvam S. De Palma R. Dany M.. (2017). TGF-β receptor I/II trafficking and signaling at primary cilia are inhibited by ceramide to attenuate cell migration and tumor metastasis. Sci. Signal. 10:eaam7464. 10.1126/scisignal.aam7464 29066540 PMC5818989

[B13] Girard D. Petrovsky N. (2011). Alström syndrome: insights into the pathogenesis of metabolic disorders. Nat. Rev. Endocrinol. 7, 77–88. 10.1038/nrendo.2010.210 21135875

[B14] Graser S. Stierhof Y. D. Lavoie S. B. Gassner O. S. Lamla S. Le Clech M.. (2007). Cep164, a novel centriole appendage protein required for primary cilium formation. J. Cell Biol. 179, 321–330. 10.1083/jcb.200707181 17954613 PMC2064767

[B15] Grgurevic L. Christensen G. L. Schulz T. J. Vukicevic S. (2016). Bone morphogenetic proteins in inflammation, glucose homeostasis and adipose tissue energy metabolism. Cytokine Growth Factor Rev. 27, 105–118. 10.1016/j.cytogfr.2015.12.009 26762842

[B16] Hearn T. (2019). ALMS1 and Alström syndrome: a recessive form of metabolic, neurosensory and cardiac deficits. J. Mol. Med. (Berl). 97, 1–17. 10.1007/s00109-018-1714-x 30421101 PMC6327082

[B17] Hearn T. Renforth G. L. Spalluto C. Hanley N. A. Piper K. Brickwood S.. (2002). Mutation of ALMS1, a large gene with a tandem repeat encoding 47 amino acids, causes Alström syndrome. Nat. Genet. 31, 79–83. 10.1038/ng874 11941370

[B18] Hearn T. Spalluto C. Phillips V. J. Renforth G. L. Copin N. Hanley N. A.. (2005). Subcellular localization of ALMS1 supports involvement of centrosome and basal body dysfunction in the pathogenesis of obesity, insulin resistance, and type 2 diabetes. Diabetes 54, 581–1587. 10.2337/diabetes.54.5.1581 15855349

[B19] Heger J. Schulz R. Euler G. (2016). Molecular switches under TGFβ signalling during progression from cardiac hypertrophy to heart failure. Br. J. Pharmacol. 173, 3–14. 10.1111/bph.13344 26431212 PMC4813390

[B20] Hernandez-Hernandez V. Pravincumar P. Diaz-Font A. May-Simera H. Jenkins D. Knight M. (2013). Bardet–Biedl syndrome proteins control the cilia length through regulation of actin polymerization. Hum. Mol. Genet. 22, 3858–3868. 10.1093/hmg/ddt241 23716571 PMC3766180

[B21] Ishikawa H. Marshall W. F. (2011). Ciliogenesis: building the cell's antenna. Nat. Rev. Mol. Cell. Biol. 12, 222–234. 10.1038/nrm3085 21427764

[B22] Kim J. Lee J. E. Heynen-Genel S. Suyama E. Ono K. Lee K.. (2010). Functional genomic screen for modulators of ciliogenesis and cilium length. Nature 464, 1048–1051. 10.1038/nature08895 20393563 PMC2929961

[B23] Knorz V. J. Spalluto C. Lessard M. Purvis T. L. Adigun F. F. Collin G. B.. (2010). Centriolar association of ALMS1 and likely centrosomal functions of the ALMS motif-containing proteins C10orf90 and KIAA1731. Mol. Biol. Cell 21, 3617–3629. 10.1091/mbc.e10-03-0246 20844083 PMC2965680

[B24] Li G. Vega R. Nelms K. Gekakis N. Goodnow C. McNamara P.. (2007). A role for Alström syndrome protein, alms1, in kidney ciliogenesis and cellular quiescence. PLoS Genet. 3:e8. 10.1371/journal.pgen.0030008 17206865 PMC1761047

[B25] Louw J. J. Corveleyn A. Jia Y. Iqbal S. Boshoff D. Gewillig M.. (2014). Homozygous loss-of-function mutation in ALMS1 causes the lethal disorder mitogenic cardiomyopathy in two siblings. Eur. J. Med. Genet. 57, 532–535. 10.1016/j.ejmg.2014.06.004 24972238

[B26] Luo K. (2017). Signaling cross talk between TGF-β/Smad and other signaling pathways. Cold Spring Harb. Perspect. Biol. 9:a022137. 10.1101/cshperspect.a022137 27836834 PMC5204325

[B27] Marshall J. D. Maffei P. Collin G. B. Naggert J. K. (2011). Alström syndrome: genetics and clinical overview. Curr. Genomics 12, 225–235. 10.2174/138920211795677912 22043170 PMC3137007

[B28] Massagué J. (2012). TGFβ signaling in context. Nat. Rev. Mol. Cell Biol. 13, 616–630. 10.1038/nrm3434 22992590 PMC4027049

[B29] Meng X. M. Huang X. R. Chung A. C. Qin W. Shao X. Igarashi P.. (2010). Smad2 protects against TGF-β/Smad3-mediated renal fibrosis. J. Am. Soc. Nephrol. 21, 1477–1487. 10.1681/ASN.2009121244 20595680 PMC3013519

[B30] Mitchison H. M. Valente E. M. (2017). Motile and non-motile cilia in human pathology: from function to phenotypes. J. Pathol. 241, 294–309. 10.1002/path.4843 27859258

[B31] Mokrzan E. M. Lewis J. S. Mykytyn K. (2007). Differences in renal tubule primary cilia length in a mouse model of Bardet-Biedl syndrome. Nephron. Exp. Nephrol. 106, e88–96. 10.1159/000103021 17519557

[B32] Pedersen L. B. Mogensen J. B. Christensen S. T. (2016). Endocytic control of cellular signaling at the primary cilium. Trends Biochem. Sci. 41, 784–797. 10.1016/j.tibs.2016.06.002 27364476

[B33] Pfaffl M. W. (2001). A new mathematical model for relative quantification in real-time RT-PCR. Nucleic Acids Res. 29:e45. 10.1093/nar/29.9.e45 11328886 PMC55695

[B34] Reiter J. F. Leroux M. R. (2017). Genes and molecular pathways underpinning ciliopathies. Nat. Rev. Mol. Cell Biol. 18:533–547. 10.1038/nrm.2017.60 28698599 PMC5851292

[B35] Shenje L. T. Andersen P. Halushka M. K. Lui C. Fernandez L. Collin G. B.. (2014). Mutations in Alström protein impair terminal differentiation of cardiomyocytes. Nat. Commun. 5:3416. 10.1038/ncomms4416 24595103 PMC3992616

[B36] Tammachote R. Hommerding C. J. Sinders R. M. Miller C. A. Czarnecki P. G. Leightner A. C.. (2009). Ciliary and centrosomal defects associated with mutation and depletion of the Meckel syndrome genes MKS1 and MKS3. Hum. Mol. Genet. 8:3311–3323. 10.1093/hmg/ddp272 19515853 PMC2733821

[B37] Vestergaard M. L. Awan A. Warzecha C. B. Christensen S. T. Andersen C. Y. (2016). Immunofluorescence microscopy and mrna analysis of human embryonic stem cells (HESCS) including primary cilia associated signaling pathways. Methods Mol. Biol. 1307, 123–140. 10.1007/7651_2014_127 25304206

[B38] Walton K. L. Johnson K. E. Harrison C. A. (2017). Targeting TGF-β mediated SMAD signaling for the prevention of fibrosis. Front. Pharmacol. 8:461. 10.3389/fphar.2017.00461 28769795 PMC5509761

[B39] Zamani N. Brown C. W. (2011). Emerging roles for the transforming growth factor-{beta} superfamily in regulating adiposity and energy expenditure. Endocr. Rev. 32, 387–403. 10.1210/er.2010-0018 21173384 PMC3365795

[B40] Zhang Y. Alexander P. B. Wang X. F. (2017). TGF-β family signaling in the control of cell proliferation and survival. Cold Spring Harb. Perspect. Biol. 9:a022145. 10.1101/cshperspect.a022145 27920038 PMC5378054

[B41] Zulato E. Favaretto F. Veronese C. Campanaro S. Marshall J. D. Romano S.. (2011). ALMS1-deficient fibroblasts over-express extra-cellular matrix components, display cell cycle delay and are resistant to apoptosis. PLoS ONE 6:e19081. 10.1371/journal.pone.0019081 21541333 PMC3082548

