# Supplementary material for: ALMS1 Regulates TGF-β Signaling and Morphology of Primary Cilia
Source: Front Cell Dev Biol. 2021 Feb 1;9:623829. doi: 10.3389/fcell.2021.623829 (PMC7882606; doi:10.3389/fcell.2021.623829)
Supplement: Supplementary file 1 [file Table_1.DOCX]

Supplementary Material

# Supplementary Figures and Tables

## Supplementary Tables

**Supplementary Table 1.** List of SMAD transcription factor binding sites in *ALMS1* promoter. Putative binding sites for SMAD transcription factors located within ALMS1 promoter were identified with Genomatix´s MatInspector software. *Matrix similarity* score reaches a maximum value of 1 only if the candidate sequence corresponds to the most conserved nucleotide for each matrix position. The core sequence used by *MatInspector* is shown in capital letters. HGNC (HUGO Gene Nomenclature Committee; http://www.genenames.org/ ).

| **TRANSCRIPTION FACTOR** | **SYMBOL**  **(HGNC ID)** | **MATRIX SIMILARITY** | **STRAND** | **SEQUENCE** |
| --- | --- | --- | --- | --- |
| SMAD family member 4 | SMAD4 (6770) | 0.995 | + | ttgGTCTaggc |
| SMAD family member 3 | SMAD3 (6769) | 0.997 | + | ggtGTCTggtt |

## Supplementary Figures


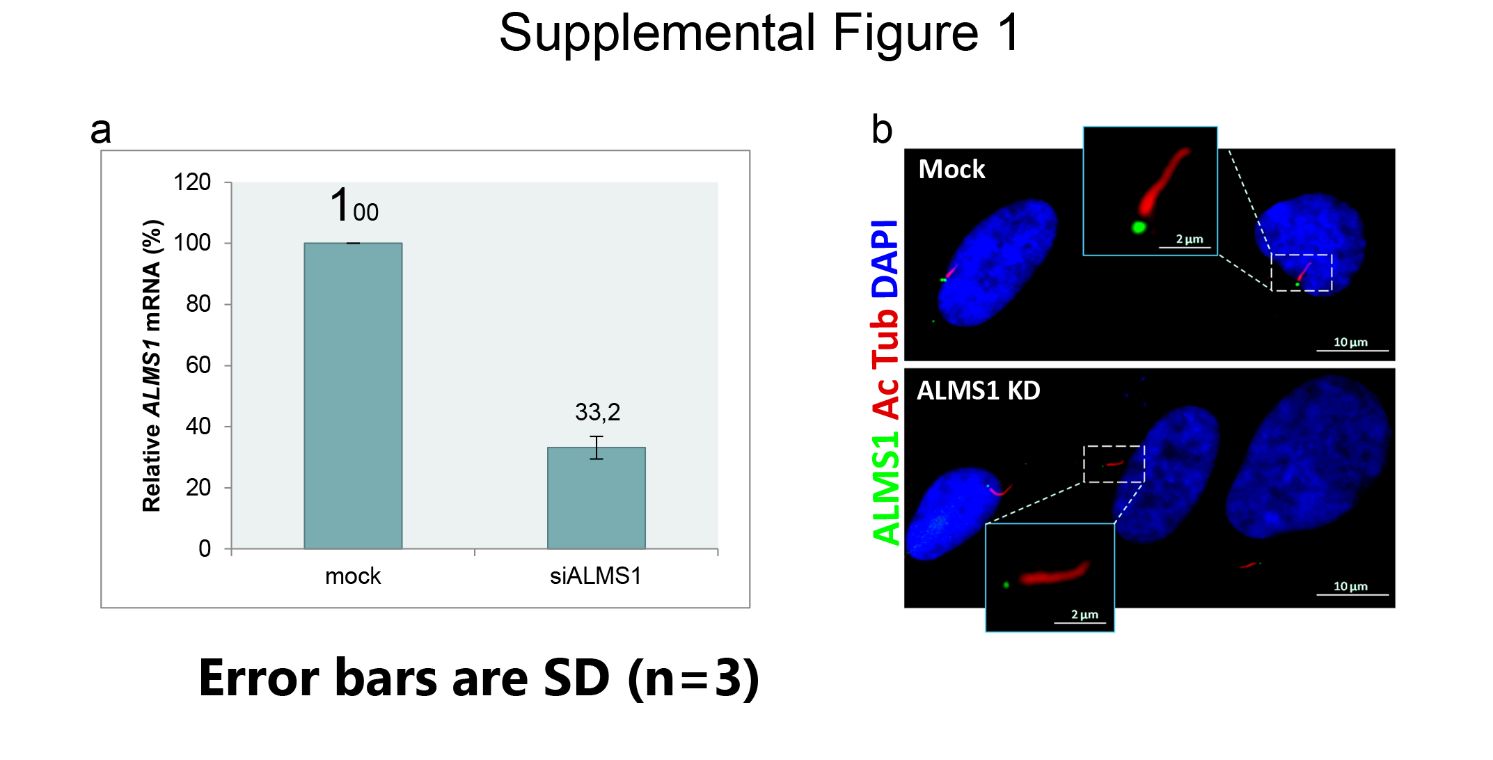


**Supplementary Figure 1.** ALMS1 knockdown validation. (a) Relative expression level of *ALMS1* mRNA in the silenced samples (siALMS1) compared with the control samples (mock). Error bars show mean ± SD (n=3). (b) Relative quantification of ALMS1 fluorescence at ciliary base in control (mock) s and silenced (siALMS1) cells. Error bars show mean ± SD (n=3).
